# Aberrant CBFA2T3B gene promoter methylation in breast tumors

**DOI:** 10.1186/1476-4598-3-22

**Published:** 2004-08-10

**Authors:** Anthony J Bais, Alison E Gardner, Olivia LD McKenzie, David F Callen, Grant R Sutherland, Gabriel Kremmidiotis

**Affiliations:** 1 Bionomics Limited, Thebarton, Adelaide, SA 5031, Australia; 2 Department of Haematology and Genetic Pathology, Flinders University, Bedford Park, Adelaide, SA 5042, Australia; 3 Department of Cytogenetics and Molecular Genetics, Women's and Children's Hospital, North Adelaide, Adelaide, SA 5006, Australia; 4 Dame Roma Mitchell Cancer Research Labs, Hanson Institute, Adelaide, SA 5000, Australia; 5 Department of Paediatrics, University of Adelaide, Adelaide, SA 5005, Australia

## Abstract

**Background:**

The CBFA2T3 locus located on the human chromosome region 16q24.3 is frequently deleted in breast tumors. CBFA2T3 gene expression levels are aberrant in breast tumor cell lines and the CBFA2T3B isoform is a potential tumor suppressor gene. In the absence of identified mutations to further support a role for this gene in tumorigenesis, we explored whether the CBFA2T3B promoter region is aberrantly methylated and whether this correlates with expression.

**Results:**

Aberrant hypo and hypermethylation of the CBFA2T3B promoter was detected in breast tumor cell lines and primary breast tumor samples relative to methylation index interquartile ranges in normal breast counterpart and normal whole blood samples. A statistically significant inverse correlation between aberrant CBFA2T3B promoter methylation and gene expression was established.

**Conclusion:**

CBFA2T3B is a potential breast tumor suppressor gene affected by aberrant promoter methylation and gene expression. The methylation levels were quantitated using a second-round real-time methylation-specific PCR assay. The detection of both hypo and hypermethylation is a technicality regarding the methylation methodology.

## Background

Allelic loss of heterozygosity (LOH) of the human chromosome 16q in several sporadic cancer types, including breast, prostate and ovary cancers, suggests this chromosome arm harbors tumor suppressive loci [1-3]. The most frequent region of allelic loss occurs within 3-megabases (Mb) at 16q24.3 between the marker D16S498 and the telomere [1,4]. Sequencing of the 3-Mb region has identified approximately 100 genes [4]. Eight of these have been excluded as potential tumor suppressors for breast cancer based on mutation analysis in tumor DNA [5]. Recently, a messenger RNA (mRNA) expression survey was completed within 2.4-Mb of this region examining the expression profiles of over 75 genes in a panel of breast tumor cell lines [6]. It was found that only three genes exhibited significantly aberrant expression profiles. These genes were highly expressed in some cell lines and lowly expressed in others. This led to the hypothesis that this aberrant expression may reflect a role for these genes as tumor suppressors in determining cancer phenotype and behavior. One of these genes was the core-binding factor, alpha subunit 2, translocation to 3; termed CBFA2T3.

**Figure 1:**
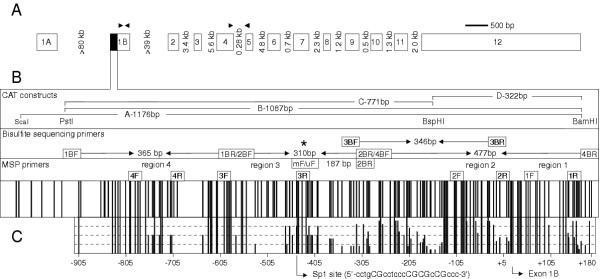
**CBFA2T3 gene structure and promoter methylation patterns. **(A) CBFA2T3 encodes two alternative transcripts, CBFA2T3A and CBFA2T3B. CBFA2T3A is encoded by exons 1A and 2–12. CBFA2T3B is encoded by exons 1B-12 splicing out exon 3. Relative exon sizes are shown. The exon 1A start site contains no CpG island. The black box marks the location of a high-density CpG island located five prime to the exon 1B start site. The black arrowheads mark the primers used for real-time RT-PCR. (B) CBFA2T3B contains a CpG island of approximately 160 CpG sites spanning 1-kb of sequence. The single black bars represent CpG sites scaled relative to each other. CAT ELISA promoter constructs and primers used for MSP, real-time MSP and bisulfite sequencing are shown. The asterisk marks the location of the amplicon and internal primers used for second-round real-time MSP. (C) CBFA2T3B promoter methylation patterns were examined in hypermethylated cell lines using sodium bisulfite sequencing. A characteristic sinusoidal pattern of approximately six high to low frequency methylation levels every 40–150 bp was detected. The high-frequency cytosine methylation levels residing within a consensus Sp1 binding site located approximately minus 450 bp from the transcriptional start of exon 1B are shown.

CBFA2T3 encodes for a protein that belongs to the eight-twenty-one (ETO) family, which also includes the genes CBFA2T1, CBFA2T2 in mammalian cells and nervy in Drosophila [7]. The mammalian members of this protein family are involved in therapy-related chromosomal translocations causing acute myeloid leukemia [8]. The CBFA2T3 gene encodes two alternative transcripts, CBFA2T3A (NM_005187) and CBFA2T3B (NM_175931) (Figure 1A). CBFA2T3A reportedly functions as a nuclear transcriptional co-repressor via its interaction with histone deacetylase (HDAC) complexes [9]. CBFA2T3B functions as a kinase anchorage protein in T lymphocytes and may play a role in inflammatory response [10]. Recently, it was demonstrated that CBFA2T3B functions as a transcriptional repressor and exhibits in vitro characteristics consistent with tumor suppressor activity [11]. This gene was found to be lowly expressed in a number of breast tumor cell lines and upon re-introduction it reduced their growth on plastic and in soft agar.

Mutation analysis in breast tumor cell lines and primary breast tumor samples failed to identify any CBFA2T3 sequence aberrations [11]. It was recognized that aberrant promoter methylation might be the mechanism responsible for the altered expression of CBFA2T3 in breast tumors. The expression of several tumor suppressor genes has been found inactivated or reduced in tumors in association with promoter hypermethylation [12]. Promoter hypermethylation can occur in conjunction with allelic loss and or mutation and is regarded as an alternative form of 'knockout' in biallelic inactivation. Accumulating evidence now suggests that promoter hypermethylation may affect genes that reside within regions of frequent allelic loss more often than mutation [13]. Alternatively, several oncogenes have been found to be over-expressed in tumors in association with promoter hypomethylation [14]. As such, aberrant promoter methylation is considered a fundamental process in developing cancers and has recently received considerable interest as a rapid non-invasive molecular screening tool for the early detection of tumor cells in a range of bodily fluids and biopsy specimens [15].

In this study, the methylation status of a high-density CpG island promoter region located five prime to the exon 1B sequence of the CBFA2T3B transcript is described (Figure 1A). We explored whether this region is aberrantly methylated in breast tumor cell lines and primary breast tumor samples and whether a correlation exists between methylation and gene expression. Both aberrant hypo and hypermethylation levels were detected in breast tumors in correlation with elevated and reduced expression. The phenomenon of hypo and hypermethylation relates to the amount of DNA used in the methylation methodology as discussed.

## Results

### Isoform-specific analysis of CBFA2T3 gene expression levels

**Figure 2:**
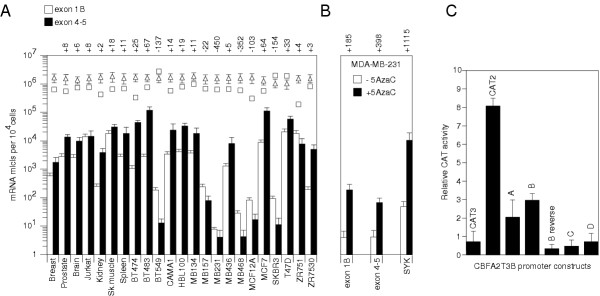
**CBFA2T3 gene expression levels, 5-Aza-dC re-expression and promoter activity. **(A) CBFA2T3 gene expression levels were assayed using real-time RT-PCR. Several breast tumor cell line and normal tissue sample expression levels are shown. The y-axis represents mRNA mlcls expressed per 10^4 ^cells shown on a log scale (mean ± SD, n = 6). Fold changes in expression relative to normal breast expression are shown above each sample. The white diamonds and white squares represent expression levels of the housekeeping genes cyclophilin A (CYPA) and ATPase coupling factor 6 subunit (ATP5A), respectively. CBFA2T3B and CBFA2T3 expressed at endogenously low yet aberrant levels in breast tumor cell lines. Using normal breast as a reference, CBFA2T3B (600 mRNA mlcls per 10^4 ^cells) and CBFA2T3 (1,800) were low compared to ATP5A (600,000) and CYPA (1,600,000). CBFA2T3 expression ranged 30,000-fold from 4 to 120,000 mRNA mlcls per 10^4^cells in MDA-MB-231 and BT-483, respectively. In contrast, CYPA and ATP5A expression ranged 2-fold and 20-fold, respectively (100–200 and 15–300 mRNA mlcls per cell). Expression levels were also examined in several primary breast tumor samples for which total RNA was available (see Figure 6). (B) CBFA2T3 re-expression levels were examined in MDA-MB-231 cells using 5-Aza-dC. Fold changes in CBFA2T3B, CBFA2T3 and SYK expression levels are shown upon exposure to 5-Aza-dC relative to control untreated cells. > 100-fold re-expression was detected in CBFA2T3 and CBFA2T3B. 5-Aza-dC had no affect on CBFA2T3A expression (data not shown). > 1,000-fold re-expression was also detected in SYK, a control gene known to be hypermethylated and down-regulated in MDA-MB-231 cells. (C) CBFA2T3B promoter activity was assayed using CAT ELISA. Promoter constructs labeled A to D (see Figure 1B) were inserted upstream of the CAT reporter in pBLCAT3 (Boehringer Mannheim). pBLCAT2 driven by the tyrosine kinase promoter was used as a positive control. 2.0 × 10^6 ^293T cells were transfected in triplicate using Lipofectamine 2000 (Invitrogen) with 1.5 μg of construct and control vector and 0.3 μg of the internal pSVβ-galactosidase control vector (Stratagene). Cells were lysed after 24 h and CAT concentrations determined using ELISA. Of the four constructs labeled A to D, the 1-kb B construct promoted a 30-fold increase in CAT expression (mean ± SD are triplicates, n = 3).

CBFA2T3 encodes two alternative transcripts, CBFA2T3A and CBFA2T3B (Figure 1A). It was recently demonstrated that CBFA2T3 expression levels are aberrant in breast tumor cell lines. In this study, the total expression levels were assayed using real-time RT-PCR and primers that span exons 4–5 of the CBFA2T3 gene. The tumor suppressor activity previously shown for CBFA2T3B, led us to examine the expression profile of this transcript in breast tumor cell lines using a TaqMan probe specific for exon 1B. CBFA2T3B displayed aberrant expression similar to the total (Figure 2A). This aberrant expression was found to be endogenously low in all samples examined. The expression of this gene was so low that it could not be reliably detected using Northern Blots or RNase protection (data not shown). In addition, expression levels of the CBFA2T3A transcript were assayed in breast tumor cell lines using a TaqMan probe specific for exon 1A. CBFA2T3A expressed at lower levels than CBFA2T3B, but due to the identification of several complex splice variants between exon 1A and exons 1B, 2, 3 and 4, further analysis is required (data not shown). An example of the raw data real-time RT-PCR expression analysis of the CBFA2T3 transcripts is shown (Additional file 1).

### Qualitative analysis of CBFA2T3B promoter methylation levels

**Figure 3:**
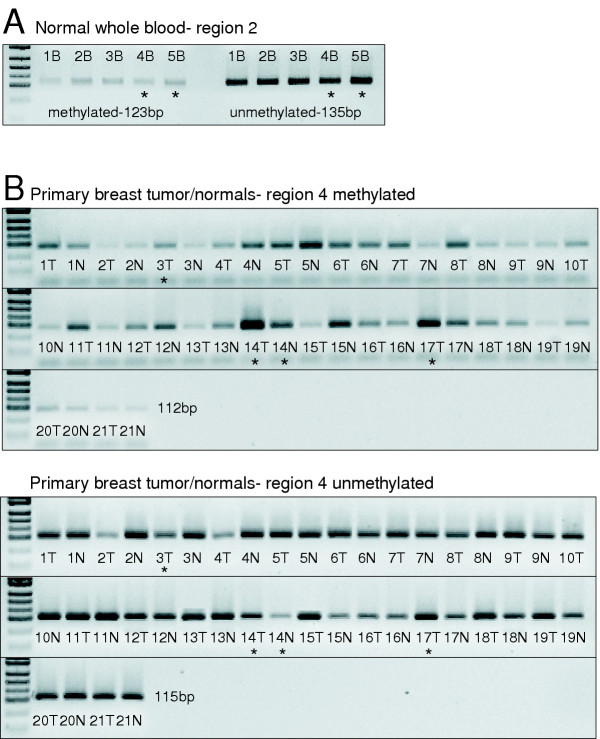
**CBFA2T3B promoter methylation levels examined using MSP. **The full results of this analysis in breast tumor cell lines, primary breast tumors, normal breast counterparts and normal whole blood samples are summarized in Additional file 2. (A) An example of the characteristic basal methylation levels detected in all samples is shown for normal blood samples examined at region 2. (B) An example of the complex high to low methylation levels is shown for 20 primary breast tumor samples with adjacent normal breast counterparts. The pUC19 DNA/MspI marker concentrations reflect an approximate concentration of 100 to 1 unmethylated to methylated mlcls. The asterisks indicate the samples examined by bisulfite sequencing.

Based on the idea that this altered expression might develop via aberrant methylation, the promoter activity and methylation status of a high-density CpG island located five prime to the exon 1B sequence of CBFA2T3B was examined (Figures 1A and 1B). The promoter activity was assayed using chloramphenicol acetyl-transferase (CAT) ELISA and confirmed that a 1-kb region spanning the island was capable of promoting a 30-fold increase in CAT expression (Figure 2C). To determine if this promoter region is aberrantly methylated in breast tumors, 24 breast tumor cell lines, 20 primary breast tumors, 20 normal breast counterparts and 24 normal whole blood samples were screened using methylation-specific PCR (MSP). Four separate 100–200 bp regions spanning the promoter were amplified using primers specific for either unmethylated or methylated cytosines at the CpG sites shown (Figure 1B). The full results of this analysis are summarized in Additional file 2. In general, a low 'basal' level of methylation was detected in all samples at the various regions examined. An example of this basal methylation is shown for the normal blood samples at region two (Figure 3A). Unlike the bloods, several breast tumor cell lines, primary breast tumors and normal breast counterpart samples displayed complex high to low methylation levels. An example of this complex methylation is shown for the primary breast tumors and their normal counterparts at region four (Figure 3B). This analysis revealed that only few cell lines displayed clear hypermethylation (e.g. MDA-MB-231) or hypomethylation (e.g. BT-483) in association with reduced and elevated expression. To support this association, it was found that treatment of MDA-MB-231 cells with the demethylating agent 5-aza-2'-deoxycytidine (5-Aza-dC) was capable of increasing both CBFA2T3 and CBFA2T3B expression levels by > 100-fold relative to controls (Figure 2B). Overall, however, it was difficult to comprehend this complex promoter methylation and an association with expression thus suggesting that further analysis was required.

### Bisulfite sequence analysis of CBFA2T3B promoter methylation patterns

**Figure 4:**
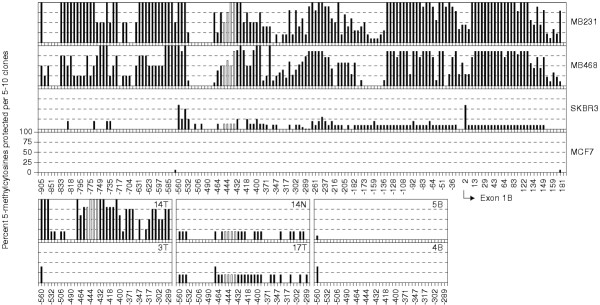
**CBFA2T3B promoter methylation patterns examined using bisulfite sequencing. **The methylation patterns in breast tumor cell lines, primary breast tumors, normal breast counterparts and normal whole blood samples are shown. The y-axis represents protected 5-methylcytosines scored as percent cytosines methylated per 5–10 clones. The complete methylation maps displaying 160 CpG sites spanning 1-kb of sequence are shown for breast tumor cell lines only. The white bars indicate the Sp1 sites targeted by second-round real-time MSP. Bisulfite sequencing of the CBFA2T3B promoter region in hypermethylated breast tumor samples MDA-MB-231, MDA-MB-468 and 14T revealed a characteristic sinusoidal methylation pattern. This sinusoidal pattern was also detected in samples SK-BR-3, 14N and 17T at levels approximately 1 tenth of the hypermethylated samples. No methylation was detected in the normal blood samples 4B and 5B or the breast tumor samples 3T and MCF-7.

To understand this complex promoter methylation, sodium bisulfite sequencing of the hypermethylated breast tumor samples was used to examine the pattern and frequency of methylation with this region. This analysis revealed that specific cytosines appeared more susceptible to methylation compared to others (Figure 4). A characteristic sinusoidal pattern of approximately six high to low frequency methylation levels every 40–150 bp was detected in cell lines and tumor samples (Figures 1C and 4). Based on this pattern, primers were designed specific for the real-time MSP quantitation of high-frequency cytosine methylation levels residing within a consensus Specificity protein (Sp1) binding site located approximately minus 450 bp from the transcriptional start of exon 1B (Figures 1B and 1C). As Sp1 proteins are commonly known to regulate gene transcription, it was considered that variable methylation at this site may be reflective of elevated or reduced expression and thus suitable for resolving and correlating the complex promoter methylation levels.

### Quantitative analysis of CBFA2T3B promoter methylation levels

**Figure 5:**
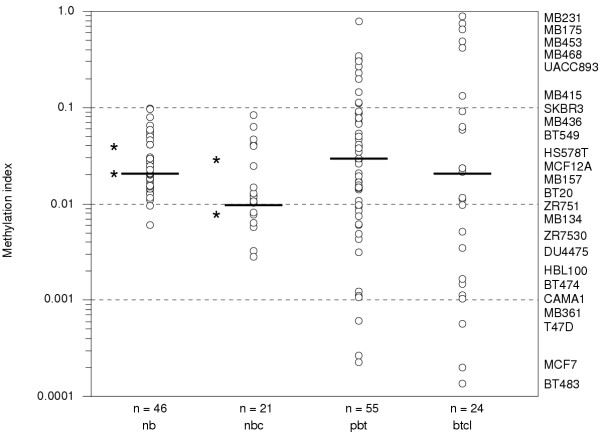
**CBFA2T3B promoter methylation levels assayed using second-round real-time MSP. **The methylation levels in normal whole blood samples (nwb), normal breast counterparts (nbc), primary breast tumors (pbt) and breast tumor cell lines (btcl) were assayed at the Sp1 site shown in Figure 1C using real-time MSP. The y-axis represents methylation levels plotted as methylation indices [mi = m/(m + u)] on a log scale. Each white circle represents a different sample. The breast tumor cell lines examined are shown in descending order from high to low methylation. The horizontal bars mark the median methylation indices calculated for each group. The asterisks mark the interquartile ranges for normal groups. The median methylation indices and (interquartile ranges) were 0.02 (0.02–0.04) for nwb, 0.01 (0.008–0.03) for nbc, 0.03 (0.009–0.08) for pbt and 0.02 (0.002–0.3) for btcl. The median methylation index variance in each tumor group was statistically significantly different than the normal groups (P = .001); nwb/btcl (P < .0001), nwb/pbt (P = .009), nbc/btcl (P = .01), nbc/pbt (P = .05). The normal group median methylation index variances were not significantly different; nwb/nbc (P = 0.6).

To assay CBFA2T3B promoter methylation levels at this Sp1 site, a bisulfite sequencing amplicon spanning this region was initially PCR amplified and column purified from 24 breast tumor cell lines, 55 primary breast tumors, 22 normal breast counterparts and 46 normal whole blood samples. Second-round real-time MSP was performed on the amplicons using internal forward primers to detect for either unmethylated or methylated cytosines at the Sp1 site. Standard curve dilutions of previously prepared internally primed clones representative of either unmethylated or methylated sequence were used to extrapolate methylation levels and normalize for differences in amplification efficiencies. The methylated cytosines were expressed as a fractional ratio of unmethylated cytosines to determine the methylation indices [mi = m/(m + u)]. On plotting the indices, a clear difference between the tumor, normal groups and complex promoter methylation levels was revealed (Figure 5). The normal blood samples maintained a specific basal methylation level and were similar to normal breast counterparts with methylation indices ranging from 0.006–0.09 and 0.002–0.08, respectively. Median methylation index levels were 0.02 in normal bloods and 0.01 in normal breast counterparts. In contrast, several breast tumor cell lines and primary breast tumors were highly variable relative to the normal samples with methylation indices ranging from 0.0002–0.8 and 0.0001–0.9, respectively. A Levene's test revealed a statistically significance difference in variances of methylation indices with the tumor groups being more varied than the normals (P = .001). It was predicted that 83–75% of breast tumor cell lines and 78–69% of primary breast tumors displayed aberrant methylation levels outside the methylation index interquartile ranges of the normal blood (0.02–0.04) and normal breast counterpart samples (0.008–0.03), respectively. Half of the aberrations detected in breast tumor cell lines were either hypo or hypermethylated relative to the normal breast counterpart interquartile ranges. Up to 22% of the primary breast tumors were hypomethylated and 47% hypermethylated relative to the normal breast counterpart interquartile ranges. An example of the raw data real-time MSP and melt curve analysis showing the aberrant methylation levels in breast tumor cell lines compared to normal whole blood samples is shown (Additional files 3 and 4).

### Correlation of CBFA2T3B promoter methylation and gene expression levels

**Figure 6:**
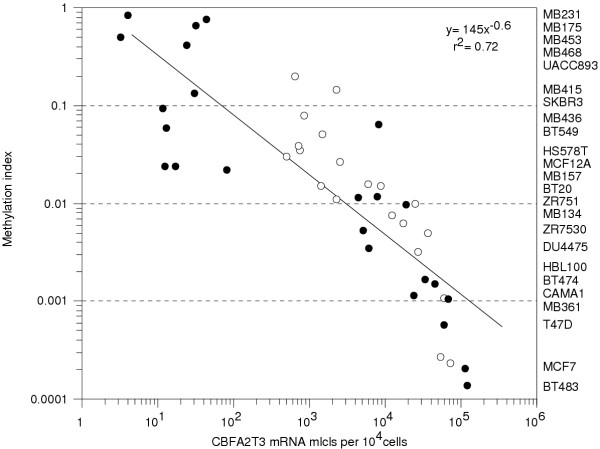
**CBFA2T3B promoter methylation levels versus gene expression. **The data for 24 breast tumor cell lines and 20 primary breast tumor samples are shown. The y-axis represents methylation levels assayed using real-time MSP and plotted as methylation indices [mi = m/(m + u)] on a log scale. The x-axis represents total expression levels assayed using real-time RT-PCR and plotted as mRNA mlcls per 10^4 ^cells on a log scale. Each white circle represents a different primary breast tumor sample and the black circles represent the breast tumor cell lines. The breast tumor cell lines examined are shown in descending order from high to low methylation. A statistically significant inverse correlation was established between promoter hypermethylation (mi = 0.9) and reduced expression (4 mRNA mlcls per 10^4 ^cells) versus hypomethylation (0.0001) and elevated expression (120,000) (r^2 ^= 0.72; r = -0.9, P < .0001). A power regression (y = cx^b^) describes the relationship between methylation and expression.

To correlate CBFA2T3B promoter methylation levels with gene expression, the methylation indices from 24 breast tumor cell lines and 20 primary breast tumor samples were plotted against their expression. A statistically significant inverse correlation between CBFA2T3B promoter methylation and exon 1B specific expression was established (r^2 ^= 0.63; r = -0.8, P = .0002). Based on the possibility that five prime RNA degradation and secondary structures may have affected the exon 1B complementary DNA (cDNA) synthesis, a correlation between methylation and the total expression is alternatively shown (Figure 6). CBFA2T3B promoter hypermethylation and reduced expression inversely correlated with hypomethylation and elevated expression (r^2 ^= 0.72; r = -0.9, P < .0001). At a hypermethylated index of around 0.9, approximately 4 mRNA molecules (mlcls) per 10^4 ^cells were detected compared to a hypomethylated index of 0.0001 and 120,000 mRNA mlcls per 10^4 ^cells. The number of CBFA2T3B promoter mlcls methylated per cell for each breast tumor cell line was also calculated by multiplying the methylation index values by the number of 16q24.3 DNA mlcls per cell as previously determined by FISH [16]. These values were plotted against expression in aim to improve the original correlation (r^2 ^= 0.77; r = -0.9, P < .0001) (Additional file 5). By plotting these data sets a power regression was derived which could be used to solve unknown x or y values.

## Discussion

In this study, it has been demonstrated that expression of the CBFA2T3B isoform is altered in breast tumors and that this correlates strongly with aberrant CpG island promoter methylation. Moreover, a comprehensive method for the detection, quantitation and correlation of promoter methylation and gene expression levels has been developed. MSP was used in combination with sodium bisulfite sequencing to identify sites within the CBFA2T3B promoter region displaying high-frequency methylation in breast tumors. Second-round real-time MSP was used to quantitate methylation levels at these sites in breast tumor cell lines, primary breast tumors, normal breast counterparts and normal whole blood samples. The CBFA2T3B promoter methylation levels were calculated as methylation indices and the indices from breast tumors were plotted against their expression.

### Validated methylation detection using the MSP and real-time MSP methods

Throughout the development of this method, it was recognized that a pre-requisite for valid MSP amplification required that sufficient amounts of bisulfite modified DNA were used in order to detect methylation and avoid stochastic effects when quantitating low target mlcl numbers and parameters relating to particle distribution statistics. The amount of DNA modified and subsequently amplified is an important parameter in terms of actually detecting potential CpG methylation in a given specimen as discussed.

Most MSP studies use 50 ng of bisulfite modified DNA for amplification. As a normal diploid cell contains an average 6.6 ρg of DNA, this equates that approximately 15,000 alleles or mlcls are made available as starting templates for MSP. If for example, 1 mlcl in 1,000 of these is methylated (mi = 0.001) for a given gene at a specific CpG site(s), then approximately 15 mlcls will act as potential starting templates. Routinely, in unmodified DNA specimens a 'purified' 100–300 bp amplicon diluted to this mlcl number should be detected at around 28–30 cycles of real-time PCR under standard primer efficiencies. Initially, this is a late cycle threshold (C_T_) for the detection of amplification and is prone to stochastic effects. Under MSP conditions, the DNA has been bisulfite modified which introduces numerous variables that further reduce the overall probability of detection. Upon modification multiple sequence permutations at the CpG site(s) of interest may arise (e.g. from 3 CpG sites a total 8 possible C to TpG permutations exist as 2^3 ^= 8). If primers are designed to detect a permutation present at say 4 mlcls in the above example of 15 mlcls (i.e. at attogram starting amounts), then under MSP, a 100–300 bp amplicon at this mlcl number will not amplify until after 35 cycles or may not amplify at all. Moreover, because the initial cycles of MSP are asymmetric the starting template mlcl numbers are further reduced. In addition, it is well known that the DNA is substantially degraded following bisulfite modification with studies demonstrating up to 84–96% degradation [17]. If 1 μg of DNA is modified, as routinely reported, and say 80% is degraded, then based on an unlikely recovery rate of 100%, only 20% of the original pool of templates used in the 50 ng will be available for amplification. Thus, in the above example if only 20% of the 15 mlcls and its possible permutations are available then they may not amplify at all. The pool of primer specific methylated templates could in fact be instantly diminished. When considering the levels of bisulfite-mediated degradation in 1 μg of DNA, even alleles methylated at levels as high as 1 in 10 might be difficult to detect or become spurious. For instance, if assuming that a 100 times more template is available (i.e. 1,500 mlcls) yet permutations exist (i.e. 400 mlcls) and the recover rate is only 4%, then as little as 16 mlcls will be available as starting templates for amplification.

Thus, several technical thresholds exist for the detection of methylation using MSP. The use of 1 μg of DNA for modification, 50 ng for amplification, sequence permutations, PCR efficiency and bisulfite-mediated DNA degradation all reduce the overall probability and validity of detection. To warrant potential detection of methylation in a given specimen at specific CpG sites above a threshold of say 1 in 1,000, it was found necessary to use at least 10X coverage of the 1 μg amount or 10 μg of DNA for modification and 400–500 ng for MSP amplification. Taking into account the above example, this should provide that at least 30–40 mlcls are made available as starting templates, which should amplify within 28 cycles. Specimens with methylation levels lower than this, such as 1 in 50,000, will not be reliably detected and require the use of second-round nested MSP amplification. In this case, the first-round will still require sufficient amounts as any initial stochastic effects may result in poor reproducibility for the second-round. Moreover, as demonstrated in this study, bisulfite sequence analysis of the CBFA2T3B promoter methylation patterns in the hypermethylated MSP samples indicated that the potential for variable methylation frequencies do exist. In this case, it was found that even with sufficient amounts of DNA the qualitative MSP was unreliable and presented complex methylation data. The use of MSP primers for sites that are not methylated or methylated at low to high frequencies, in combination with these other technical thresholds, created complex stochastic methylation data merely decipherable by examining several regions. As a result, this concealed the true methylation status in a majority of the samples under investigation. Only those samples with clear hypermethylation (e.g. MDA-MB-231) or hypomethylation (e.g. BT-483) were greatly reproducible and associated with reduced and elevated expression (Figure 2A and Additional file 2).

To overcome these technical thresholds, a second-round real-time MSP assay was developed. A sequencing amplicon displaying high-frequency methylation sites was initially PCR amplified under 10X coverage conditions and column purified from all samples. Second-round real-time MSP was performed on approximately 100 ρg of the amplicon using internal forward primers to detect for either unmethylated or methylated cytosines at the high-frequency sites. Using this approach, methylation was detectable in every sample examined. All samples amplified with 28 cycles and a methylation index was calculated for these. In comparison, when the standard MSP conditions were assayed by real-time using SYBR Green I, it was found that the C_T _of methylation detection was below 28 cycles for most samples (data not shown). Extremely low methylation levels, such as those in BT-483, were undetectable using this method but were detected using the second-round. In the latter case, up to 1 methylated mlcl in 10,000 (mi = 0.0001) could be reliably detected within 25 cycles of amplification (Figure 5 and Additional file 3). Notably, no wild-type, unmethylated or methylated cross-amplification was detected when using more DNA. If this occurs then there may be a problem with primer design or modification as the conversion should be complete. In fact, with more DNA a bias in amplification for the target sequence should be created.

In addition, several other technicalities were overcome. The use of only a single second-round primer to quantitate the methylation levels of the high-frequency sites limited the number of possible 5-methyl-CpG sequence permutations that could perturb the accuracy of quantitation. This coupled with the quantitation of methylation levels from absolute standard curves enabled the normalization of reaction efficiencies and calculation of absolute methylation indices [mi = m/(m + u)] and absolute methylation ratios (u/m). The methylation ratios are simply an alternative way of expressing the methylation data (Additional file 6). The calculation of these single methylation values based on the quantitative normalization discriminates against biased amplifications and comparisons of unmethylated to methylated 'band' intensities when using MSP. Moreover, the absolute quantitation of indices and ratios offers improvement over the relative methods, such as comparative cycle thresholds (ΔΔC_T_), as they have biological significance, are less consuming, more accurate, and do not require the dynamic range in amplification efficiencies of target and references to be similar to enable valid quantitation.

### The concept of gene promoter hypo and hypermethylation

Thus, by using this method it was possible to detect CBFA2T3B promoter methylation in all samples. This phenomenon may actually be widespread for genes under the control of methylation as in accordance with the replication model of maintenance methylation [18-20]. This model might suggest that the CpG island is not just randomly methylated; the CpG island is 'always' methylated by memory yet propagated at variable levels for cell type-specific expression or at aberrant levels in association with cancers. Remarkably, in this study it was found that the methylated CBFA2T3B CpG island is propagated as a sinusoidal pattern at aberrant levels in both permanent cell lines and recently resected tumor specimens suggesting that a methylation memory does exist. In fact, it was recognized that the bisulfite sequencing detection levels of this sinusoidal pattern complemented the second real-time MSP quantitations. For example, a methylation ratio in SK-BR-3 of 10:1 (mi = 0.1) was concordant with a 1 in 10 or 10% 5-methylcytosine per 10 clone frequency (Figure 4 and Additional file 6). If by comparing the other bisulfite sequencing levels with their methylation ratios this might suggest that up to 30, 60, 90 or 5,000 clones of 4B, 5B, 3T and MCF-7, respectively, would require sequencing in order to detect 1 methylated sinusoidal mlcl.

Based on the idea that promoter methylation may occur ubiquitously, the concept arises; what is hypo and hypermethylation. In this study, it was demonstrated that hypo and hypermethylation are merely a prediction of levels outside a majority or 'interquartile range' of methylation levels found in the normal samples. For instance, of all the methylation levels detected in the cell lines, only 37.5% of these were predicted as either hypo or hypermethylated relative to the interquartile ranges of normal breast methylation. Moreover, the number of predicted hypo and hypermethylated samples are much lower when compared to the 'full range' of normal methylation ratios as shown in Additional file 6. This is considerably lower for the primary breast tumor samples (i.e. only 24% hypermethylated relative to normal breast) and is likely due to the heterogeneity of breast tumors in concealing the true tumor-related methylation levels. Regardless, by using a more sensitive detection method, the phenomenon of hypo and hypermethylation has appeared.

The phenomenon hypomethylation is a reflection of the low methylation levels that can be detected in the 'hypomethylated' samples relative to the methylated and hypermethylated samples. Alternatively, the phenomenon of hypermethylation is readily observed yet indicates that the detection of methylation alone does not simply represent hypermethylation. The CBFA2T3B gene is methylated in all samples and according to MSP in a high-percentage of samples. In effect, several studies which have examined the methylation status of potential tumor suppressors and cancer-related genes often demonstrate methylation in a high-percentage of samples and state hypermethylation based on the detection of methylation. Examples include, ras-effector nore1A (RASSF1A) [21,22], stratifin (14-3-3σ) [23], p15 and p16 [24], O6-methylguanine-DNA methyltransferase (MGMT) [25], mismatch repair gene (hMLH1) [26] and hyperplastic colon polyps gene (HPP1) [27]. In these cases, as with CBFA2T3B, it is probable that only a small percentage of this methylation has cancerous significance in terms of 'methylation-induced silencing', which is routinely confirmed by correlation with 'absence' of expression. Notably, several studies use insufficient amounts of RNA and cDNA for expression analysis and state the absence of amplification to represent inactivation rather than a level of reduction, particularly in cases of low endogenous expression. The need for quantitation to classify methylation levels has been recognized for genes such as glutathione S-transferase P1 (GSTP1) [28] and adenomatous polyposis coli (APC) [29], although in these cases the use of low DNA may nevertheless affect intra and interassay reproducibility.

In biological terms, several scenarios exist as to how these aberrant methylation levels might develop. In the case of hypomethylation, a correlation could be made with severely duplicated chromosome copies of 16q24.3 (e.g. BT-483, MCF-7). A possible scenario here is that the hypomethylation is apparent because of a duplicated copy number and thus, for example, not a direct cause of reduced DNA methyltransferase activity or over-expression per se. In the case of hypermethylation, a correlation could be made with 16q24.3 LOH (e.g. MDA-MB-231, MDA-MB-468). An emerging scenario here is that the hypomethylation induces 16q LOH to promote aberrant DNA methyltransferase activity and hypermethylation [13]. Accumulating evidence suggests that the hypermethylation itself, or 'aberrant' methylation, may be targeted to constantly methylated CpG islands (i.e. methylation induces methylation), and in addition targeted to transcriptionally inert CpG islands [19]. In this study, it was found that the CBFA2T3B promoter region is in fact constantly methylated at a median methylation index of 0.02 (i.e. 2 mlcls in 100 are methylated). When comparing this median methylation index with a median gene expression index from the breast tumor cell lines, it was calculated that only approximately 20 mRNA mlcls per 98 'active' alleles are transcribed. These calculations are shown in Additional file 5. This suggests that the CBFA2T3B gene is not only constantly methylated but also largely transcriptionally inert with the remaining active alleles possibly trans-factor dependent for expression.

Although the nature of such targeted aberrations are unknown, several studies demonstrating that altered maintenance and or de novo DNA methyltransferase activities can induce tumorigenesis, clearly demonstrates that the controlled methylation of CpG islands is crucial for normal cell development [30-34]. Accordingly, an in silico prediction of several Sp1, homeotic, epidermal and insulin growth factor recognition sites within the CBFA2T3B promoter region may implicate a role for this element in epithelial development. How the CBFA2T3B CpG island is maintained and dispersed at specified levels within a population of cells is unknown but likely relates to the methylation machinery in controlling distribution within a cell type-specific population (i.e. methylation memory and or phenotype). In this study, it was shown that aberrant deviations outside these specified levels occur profoundly in a majority of breast tumors, particularly the pure tumorigenic cell lines, and as such might comprise an element in tumor formation.

## Conclusions

Overall, this study lends further support to the idea that CBFA2T3B is aberrantly regulated in breast cancer. Additional clues supporting a role for this gene in tumor suppression may reside within its protein structure. CBFA2T3B contains a characteristic zinc finger myeloid-nervy-DEAF-1 (zf-MYND) domain and nervy homology regions [7]. Several studies demonstrate that these regions function in transcriptional co-repression via interaction with HDAC and or nuclear co-repressor complexes [9,35-38]. Accumulating evidence suggests the zf-MYND domain, which is also common to developmental proteins RP-8, DEAF-1, suppressin, Blu, BS69, PDCD2 and Bop, may interact with co-repression complexes to regulate cell-cycle transcription during cell type-specific differentiation [39-46]. Abnormal regulation may be central to tumor formation as supported by reports that several zf-MYND-like proteins display tumor suppressive activity.

Recently, much emphasis has been placed on the development of methylation-based tumor biomarkers for early breast cancer detection to predict disease outcome and strategies for therapy [15]. Interesting data indicates that the use of nipple aspirate fluids may provide for a rapid non-invasive source of screening material for methylation biomarker analysis [47]. Similar studies are underway to evaluate if the methylation status of CBFA2T3B presents biomarker utility. Although in this case, because the normal breast fluids and breast tumors themselves are histologically complex tissues containing a variety of cell types, it is recognized that further studies are required to refine the methylation index interquartile ranges. This may involve large-scale comparisons between normal and tumor cells captured using laser microdissection. Ideally, such normal controls would be resected at autopsy or reduction mammoplasty from non-risk category individuals.

## Methods

### Sample collection and nucleic acid isolation

24 breast tumor cell lines were obtained from the American Type Culture Collection and cultured under recommended conditions. 46 normal whole blood samples, 55 primary breast tumor samples with pathologically classified grade III lesions and 22 adjacent normal breast counterparts samples were obtained with clinical research approval from the Flinders Medical Centre, Department of Surgery. Breast tumor cell line, primary breast tumor and normal breast counterpart genomic DNA was isolated using GenElute for Mammalian Tissues (Sigma). Whole blood genomic DNA was isolated using the QIAamp DNA Blood Kit (Qiagen). Breast tumor cell line and primary breast tumor total RNA was isolated using RNAqueous-4PCR (Ambion). Nucleic acid concentrations were determined using RiboGreen (Molecular Probes). Breast tumor cell line, primary breast tumor and normal tissue (Clontech) total RNA extracts were DNase I treated (Ambion).

### Real-time reverse transcription-PCR (RT-PCR)

10–20 μg of total RNA was oligo(dT)_16 _reverse transcribed at 55°C for 2 h using MMLV (Promega) with addition of RNAguard (Promega) and 5% DMSO. CBFA2T3, CYPA, ATP5A and SYK expression levels were assayed by real-time RT-PCR using SYBR Green I (BMA). CBFA2T3 isoform expression levels were assayed using TaqMan probes specific for exons 1A and 1B (GeneWorks). Real-time RT-PCR was performed on a Rotor-Gene 2000 (Corbett Research) using standard 25 μl HotStar Taq conditions (Qiagen) on cDNA equivalent to 100–1,500 ng total RNA. 0.35X final SYBR Green I or 200 nM probe was used for detection. Amplifications were at 95°C for 10 min, 45 cycles at 94°C for 20 s, annealing temperature for 30 s and 72°C for 30 s. Primer sequences and annealing temperatures are shown (Additional file 7). Unknown expression levels were extrapolated from standard curve dilutions of column purified cDNA amplicons. Replicate standard curve assays (n ≥ 2) were used with C_T _coefficient variations averaging < 15% over six orders within replicates and between dilutions. mRNA mlcl numbers were quantitated from samples at the parameter C_T _from standard curves with known mlcls/μl calculated from the dilution mass in μg/μl ÷ M.W. of ssRNA transcript (× 6.02 × 10^17 ^mlcls/μmole). mRNA mlcls per cell were calculated at the C_T _concentration ÷ amount of total RNA (ρg) per reaction multiplied by 4 based on a 4 ρg total RNA per cell estimation. mRNA was shown as raw expression data (n > 4) and or normalized against CYPA or ATP5A. Several other housekeeping and cancer-related gene expression levels were quantitated to ensure the mRNA mlcl per cell estimations were compatible with other methods (data not shown).

### Sodium bisulfite modification

10–20 μg of genomic DNA was digested overnight at 37°C with restriction enzymes XbaI, XhoI, HindIII and EcoRI and cleaned using nucleotide purification columns (QIAvac 24, Qiagen). The digested DNA was pooled and bisulfite modified [17]. 10 μg of DNA was diluted in 500 μl of water and denatured with 55 μl of 2 M NaOH for 20 min at 37°C. DNA was mixed with 300 μl of 10 mM hydroquinone (Sigma), 5.2 ml of 3.6 M NaHSO_3 _(pH 5.0) (Sigma), overlaid with paraffin oil and deaminated in the dark for 16 h at 55°C. DNA was desalted using Qiagen purification columns, eluted in 500 μl water and desulfonated with 55 μl of 3 M NaOH for 20 min at 37°C. DNA was neutralized and precipitated with 800 μl of 10 M ammonium acetate (pH 7.0), 20 μl linear acrylamide and 5 ml cold 100% EtOH. Modified DNA was pelleted, resuspended in 40 μl 1 mM Tris-Cl (pH 8.0) and the concentrate stored at -80°C.

### Methylation-specific PCR (MSP) and sodium bisulfite sequencing

Four 100–200 bp regions spanning approximately 1-kb of the CBFA2T3B promoter region were amplified from ≥ 300 ng of bisulfite modified DNA under standard HotStar Taq conditions using primers specific for either unmethylated or methylated cytosines. Hypermethylated breast tumor samples were used for bisulfite sequencing to generate a CBFA2T3B promoter region methylation map. Four 300–400 bp regions spanning the promoter were amplified under standard conditions as described above using primers simultaneous for both unmethylated and methylated cytosines. The amplicons were sub-cloned into pGEM (Promega) and 5 to 10 clones sequenced using BigDye (Applied Biosystems). Primer sequences and annealing temperatures are shown (Additional file 7).

### Demethylation assay

MDA-MB-231 was treated with the demethylating agent 5-Aza-dC (Sigma). Approximately 2.0 × 10^5 ^cells per T75 flask were seeded in 10 ml RPMI-1640 supplemented with 10% FCS, 15 mM HEPES, 10 mg/liter PGS and cultured for 48 h at 37°C with 5% CO_2_. Cells were treated with 50 μm 5-Aza-dC for 120 h and replenished with fresh medium and 5-Aza-dC every 24 h. These concentrations are not inhibitory to cell growth [48]. Concentrations ranging from 1–5 μM 5-Aza-dC had no affect on expression levels (data not shown). Control untreated cells were cultured in parallel and supplemented with DMSO. Replicate T75 flasks for both treatments and controls were performed (n = 4). Total RNA was isolated and analyzed for CBFA2T3 isoform and SYK expression levels using real-time RT-PCR. MDA-MB-231 cells were also treated with the HDAC inhibiting agents trichostatin A (Sigma), sodium butyrate (Sigma) and apicidin (Calbiochem). Only trichostatin A elicited re-expression levels similar to 5-Aza-dC (data not shown).

### Real-time methylation-specific PCR (MSP)

A sequencing amplicon was initially tested for bisulfite PCR amplification and cloning bias by scoring percent methylation frequencies of overlapping amplicons. Bisulfite PCR bias was also tested by amplification on proportional mixtures of hypo and hypermethylated bisulfite treated DNA. This amplicon was PCR amplified and column purified from all samples. Second-round real-time MSP was performed on the amplicon using internal forward primers to detect for either unmethylated or methylated cytosines at the Sp1 site displaying high-frequency cytosine methylation. The methylated and unmethylated primer specificities were tested by real-time amplification on serial dilutions of unmethylated and methylated clones, respectively. Second-round amplicons were also sequenced to ensure specificity. Second real-time MSP was performed on a Rotor-Gene 2000 using internal forward primers under standard 25 μl HotStar Taq conditions with approximately 100 ρg of first-round cleaned amplicon and 0.35X SYBR Green I. Amplifications were at 95°C for 10 min, 45 cycles at 94°C for 20 s, annealing temperature for 30 s and 72°C for 30 s. Unknown unmethylated and methylated cytosine levels were extrapolated at the Sp1 site from standard curve dilutions of internally PCR amplified and column purified cloned amplicons originally sequenced and found to be representative of either unmethylated (e.g. BT-483) or methylated (e.g. MDA-MB-231) sequence. Unmethylated and methylated mlcl numbers were quantitated from all samples at the parameter C_T _from standard curves with known mlcls/μl calculated from the dilution mass in μg/μl ÷ M.W. of dsDNA clone sequence (× 6.02 × 10^17 ^mlcls/μmole). CBFA2T3B promoter methylation levels were expressed as methylation indices [mi = m/(m + u)] and ratios (u/m) [49,50].

### Statistical analysis

CBFA2T3B promoter methylation index medians and interquartile ranges were determined for each group of tissue samples. Statistical comparisons between groups were performed using the two-way Levene's test for Equality of Variances, i.e. H0: median methylation index variances are the same in each group; HA: median methylation index variances are not the same in each group. Variances in the median methylation indices between each group were considered statistically significant when P ≤ .05. Correlations between CBFA2T3B promoter methylation and gene expression levels were determined by calculating a Spearman's rank coefficient. All statistical analyses were performed using GraphPad Prism Version 4.0.

## Authors' contributions

AJB completed the work and manuscript. AEG aided in bisulfite sequencing. OLDM performed promoter analysis. DFC, GRS and GK supervised the work. All authors read and approved the final manuscript.

## Supplementary Material

Additional File 1 **CBFA2T3 gene expression levels assayed using real-time RT-PCR **The raw data CBFA2T3B expression levels and preliminary CBFA2T3A expression levels in breast tumor cell lines are shown. The y-axis represents the fluorescence detection scale and the x-axis represents the C_T _of amplification. The CBFA2T3 gene expresses at endogenously low levels and requires at least 500 ng of reverse transcribed total RNA to cDNA template for reproducible detection. In contrast, the housekeeping genes such as CYPA require only 100 ng of template. When 500 ng is used, the CYPA expression levels are off the fluorescence scale. Note the C_T _values are below 35 cycles for the down-regulated cell lines such as MDA-MB-231. This low-level of expression is undetectable by conventional RT-PCR. Moreover, because of this low endogenous expression the CBFA2T3 mRNA could not be reliably detected using Northern Blots or RNase protection (pdf file). Click here for file

Additional File 2 **CBFA2T3B promoter methylation levels examined using MSP **The methylation levels in 24 breast tumor cell lines (labeled), 20 primary breast tumors (1T-20T), 20 normal breast counterparts (1N-20N) and 24 normal whole blood samples (1–24) are shown. Four separate regions (1–4) spanning 1-kb of CBFA2T3B promoter sequence were amplified using primers to detect for either unmethylated or methylated cytosines (see Figure 1B for primer locations and Additional file 7 for primer sequences and annealing temperatures). The asterisks indicate the samples examined by bisulfite sequencing. Unmethylated (U) and methylated (M) band intensities were scored as high (black dot), low (gray dot) or negative (white dot). Low-level basal methylation defined by high unmethylated to low methylated band intensities was detected in all normal bloods in ≥ 2/4 regions. High methylation was also detected in 21% of bloods in region four. Samples were considered hypermethylated when high intensity bands amplified in all four regions or hypomethylated when no methylation was detected. Based on this, 21% of breast tumor cell lines were hypermethylated (e.g. MDA-MB-231 and MDA-MB-468), 16% hypomethylated (e.g. BT-483 and MDA-MB-361) and 63% displayed a combination of basal to high methylation. Primary breast tumor samples were complex. 57% displayed basal methylation in ≥ 2/4 regions, 14% displayed high methylation in ≥ 2/4 regions and 29% tested negative in ≥ 3/4 regions. Normal breast counterpart samples were similar with 62% displaying basal methylation in ≥ 2/4 regions, 14% displaying high methylation in ≥ 2/4 regions and 24% testing negative in ≥ 3/4 regions. The detection of high-methylation in MCF-7 is due to spurious amplification events. Several overlapping primers spanning this region in this cell line were methylation negative (data not shown). This cell line is hypomethylated according to real-time MSP (pdf file). Click here for file

Additional File 3 **CBFA2T3B promoter methylation levels assayed using second-round real-time MSP **CBFA2T3B promoter methylation levels were assayed using second-round real-time MSP. The raw data methylation levels in normal whole blood samples and breast tumor cell lines are shown. The y-axis represents the fluorescence detection scale and the x-axis represents the C_T _of amplification. Second round real-time MSP was performed on a bisulfite sequencing amplicon using internal forward primers to detect for either unmethylated (uF) or methylated (mF) cytosines at the Sp1 CpG sites shown in Figure 1C. The C_T _for methylated amplification in breast tumor cell lines was highly aberrant compared to normal blood samples. Note the late unmethylated C_T _obtained in MDA-MB-231 compared the early hypermethylated C_T_. In contrast, BT-483 shows an early unmethylated and late hypomethylated C_T _(pdf file). Click here for file

Additional File 4 **CBFA2T3B promoter methylation melt curve analysis **CBFA2T3B promoter methylation melt curves were examined following second-round real-time MSP. Raw data melt curves of second round amplicons in normal blood samples and breast tumor cell lines are shown. Curves were calculated from the negative derivative in fluorescence over temperature versus temperature (-dF/dT_m _versus T_m_). Normal blood samples displayed consistent peak levels for both the unmethylated and methylated mlcls. In contrast, breast tumor cell lines displayed highly aberrant peak levels depicted by broad melt transitions and heterogeneous melt curves reflective of the aberrant concentration and composition of 5-methylcytosines (pdf file). Click here for file

Additional File 5 **CBFA2T3B promoter methylation levels versus gene expression **Methylation indices were calculated as methylated promoter mlcls per 10^4 ^cells for breast tumor cell lines with pre-determined 16q24.3 DNA mlcls per cell and plotted against CBFA2T3 mRNA mlcls per 10^4 ^cells. The y-axis represents methylation levels assayed using real-time MSP and the x-axis represents expression levels assayed using real-time RT-PCR. Both data sets are shown on a log scale. Each black circle represents a different breast tumor cell line. The asterisk marks the median methylation and median gene expression levels. The median methylation index of 0.02 (i.e. 2 mlcls or alleles methylated in 100) is calculated from the median methylation level of 450 methylated alleles per 10^4 ^cells divided by the number of unmethylated 'active' alleles in the 10^4 ^cells or 20,000 alleles, i.e. [450 ÷ (20,000 - 450) = 0.02]. The median gene expression index of 0.2 (i.e. 20 mRNA mlcls expressed in 98 'active' alleles) is calculated from the median expression level of 4,500 mRNA mlcls per 10^4 ^cells divided by the number of unmethylated 'active' mlcls in the 10^4 ^cells, i.e. (4,500 ÷ 19,550 = 0.2). This calculation equates to approximately 4–5 mRNA mlcls expressed per 10 cells and suggests that the CBFA2T3B gene is largely transcriptionally inert. The remaining active alleles may be trans-factor dependent for expression. An inverse correlation between promoter methylation and expression levels per population of cells was established (r^2 ^= 0.77; r = -0.9, P < .0001). In hypermethylated MDA-MB-231, approximately 17,000 promoter mlcls were methylated (i.e. mi = 0.85 as 17,000 in 20,000 are methylated) and 4 mRNA mlcls expressed per 10^4 ^cells. In hypomethylated BT-483, approximately 5 promoter mlcls were methylated (mi = 0.0002) and 120,000 (± 40,000) mRNA mlcls expressed per 10^4 ^cells. This elevated expression equates that 12 (± 4) mRNA mlcls per cell are expressed from an estimated four-promoter mlcls per cell (i.e. four-16q24.3 DNA mlcls per cell) or that one 'active' unmethylated CBFA2T3B promoter mlcl per cell transcribes 2–4 mRNA mlcls. From a methylation index of approximately 2% and greater, a large increase in y may lead to a small decrease in x. Under this condition, the relationship may be asymptotic. The regression equation y = 25,801 x^-0.58 ^(r^2 ^= 0.7669) describes the methylation and expression relationship. When plotted as methylation index values the equation was y = 1.05 x^-0.61 ^(r^2 ^= 0.7665) (pdf file). Click here for file

Additional File 6 **CBFA2T3B promoter methylation levels assayed using second-round real-time MSP **The methylation levels in normal whole blood samples, normal breast counterparts, primary breast tumors and breast tumor cell lines were assayed at the Sp1 site shown in Figure 1C and plotted as absolute methylation ratios (u/m). All samples are labeled corresponding in part to Additional file 2. The asterisks indicate the samples examined by bisulfite sequencing. The gray highlights indicate the normal blood and normal breast counterpart 'full' methylation ranges. Basal methylation ratios in normal bloods averaged 60:1 (cumulative mean) unmethylated to methylated CBFA2T3B promoter mlcls. This average coincided with conventional MSP band intensities of 100:1 based on pUC19 DNA/MspI marker concentrations (see Figure 3) and the median methylation index of 0.02 (i.e. 2 mlcls in 100 are methylated). Normal blood ratios ranged 20:1 to 160:1 unmethylated to methylated mlcls. Normal breast counterparts were similar to normal bloods averaging 100:1 but with a larger range of 10:1 to 350:1. Relative to the normal samples, breast tumors displayed highly aberrant methylation ratios clearly resolved by second-round real-time MSP. 75% of breast tumor cell lines were aberrantly methylated outside the full range of normal blood basal methylation. 58% were outside the range of normal breast. Half of the aberrations were either hypo or hypermethylated relative to both normal blood and normal breast. Similar to cell lines, 51% of primary breast tumors were aberrantly methylated relative to normal blood. 35% were aberrant relative to normal breast (i.e. 24% were hypermethylated and 11% hypomethylated). Aberrant methylation ratios ranged from 1:10 in hypermethylated MDA-MB-231 to 7,000:1 in hypomethylated BT-483 (pdf file). Click here for file

Additional File 7 **Primers, probes and annealing temperatures used **(pdf file). Click here for file
