# Supplementary material for: Aberrant CBFA2T3B gene promoter methylation in breast tumors
Source: Mol Cancer. 2004 Aug 10;3:22. doi: 10.1186/1476-4598-3-22 (PMC516017; doi:10.1186/1476-4598-3-22)

Additional file 1. Real-time RT-PCR demonstrating low abundance mRNA inherent to CBFA2T3 transcripts

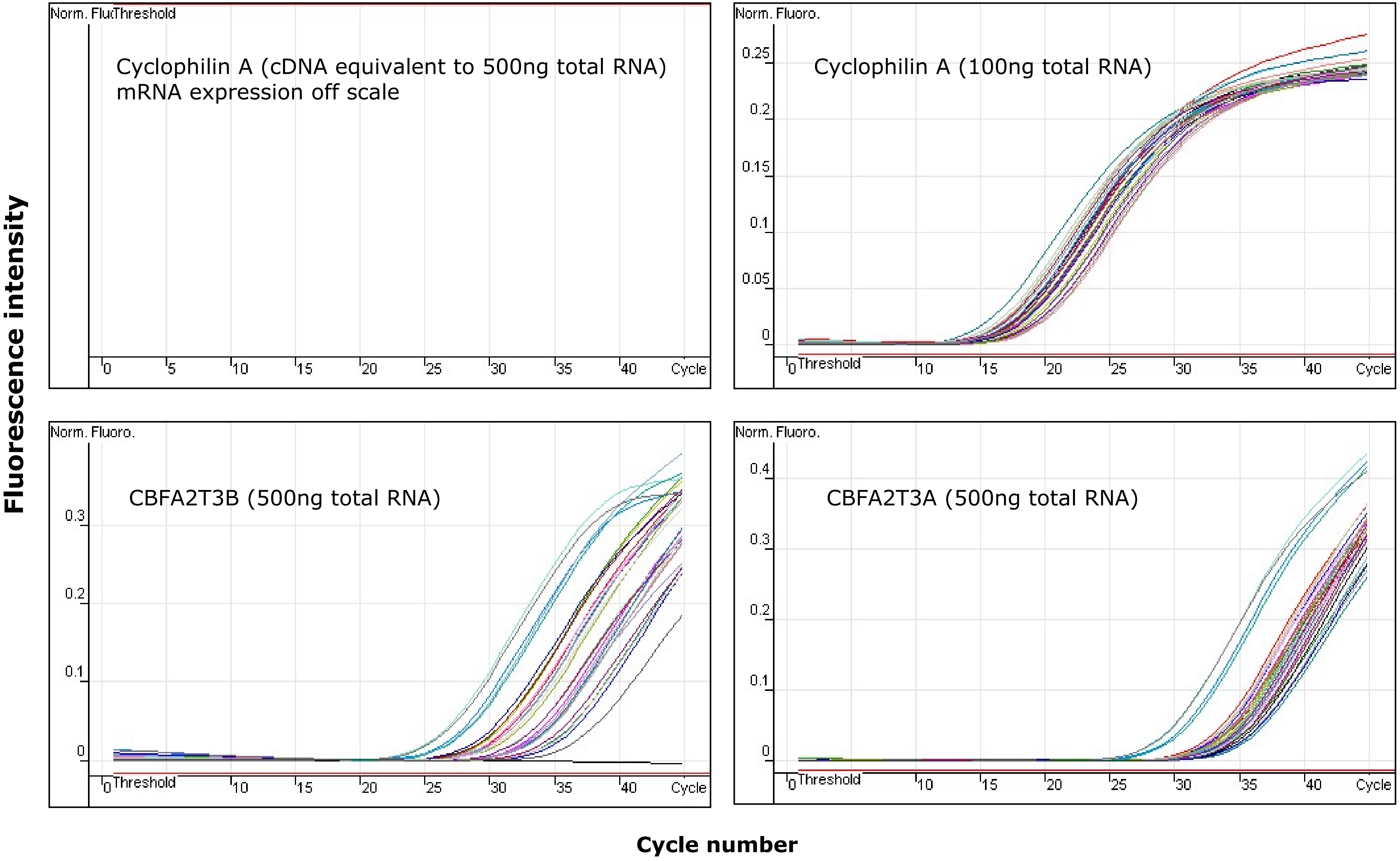

Supplement: Additional File 1 — CBFA2T3 gene expression levels assayed using real-time RT-PCR The raw data CBFA2T3B expression levels and preliminary CBFA2T3A expression levels in breast tumor cell lines are shown. The y-axis represents the fluorescence detection scale and the x-axis represents the CT of amplification. The CBFA2T3 gene expresses at endogenously low levels and requires at least 500 ng of reverse transcribed total RNA to cDNA template for reproducible detection. In contrast, the housekeeping genes such as CYPA require only 100 ng of template. When 500 ng is used, the CYPA expression levels are off the fluorescence scale. Note the CT values are below 35 cycles for the down-regulated cell lines such as MDA-MB-231. This low-level of expression is undetectable by conventional RT-PCR. Moreover, because of this low endogenous expression the CBFA2T3 mRNA could not be reliably detected using Northern Blots or RNase protection (pdf file). [file 1476-4598-3-22-S1.pdf]
