# Supplementary material for: Aberrant CBFA2T3B gene promoter methylation in breast tumors
Source: Mol Cancer. 2004 Aug 10;3:22. doi: 10.1186/1476-4598-3-22 (PMC516017; doi:10.1186/1476-4598-3-22)
Supplement: Additional File 2 — CBFA2T3B promoter methylation levels examined using MSP The methylation levels in 24 breast tumor cell lines (labeled), 20 primary breast tumors (1T-20T), 20 normal breast counterparts (1N-20N) and 24 normal whole blood samples (1–24) are shown. Four separate regions (1–4) spanning 1-kb of CBFA2T3B promoter sequence were amplified using primers to detect for either unmethylated or methylated cytosines (see Figure 1B for primer locations and Additional file 7 for primer sequences and annealing temperatures). The asterisks indicate the samples examined by bisulfite sequencing. Unmethylated (U) and methylated (M) band intensities were scored as high (black dot), low (gray dot) or negative (white dot). Low-level basal methylation defined by high unmethylated to low methylated band intensities was detected in all normal bloods in ≥ 2/4 regions. High methylation was also detected in 21% of bloods in region four. Samples were considered hypermethylated when high intensity bands amplified in all four regions or hypomethylated when no methylation was detected. Based on this, 21% of breast tumor cell lines were hypermethylated (e.g. MDA-MB-231 and MDA-MB-468), 16% hypomethylated (e.g. BT-483 and MDA-MB-361) and 63% displayed a combination of basal to high methylation. Primary breast tumor samples were complex. 57% displayed basal methylation in ≥ 2/4 regions, 14% displayed high methylation in ≥ 2/4 regions and 29% tested negative in ≥ 3/4 regions. Normal breast counterpart samples were similar with 62% displaying basal methylation in ≥ 2/4 regions, 14% displaying high methylation in ≥ 2/4 regions and 24% testing negative in ≥ 3/4 regions. The detection of high-methylation in MCF-7 is due to spurious amplification events. Several overlapping primers spanning this region in this cell line were methylation negative (data not shown). This cell line is hypomethylated according to real-time MSP (pdf file). [file 1476-4598-3-22-S2.pdf]

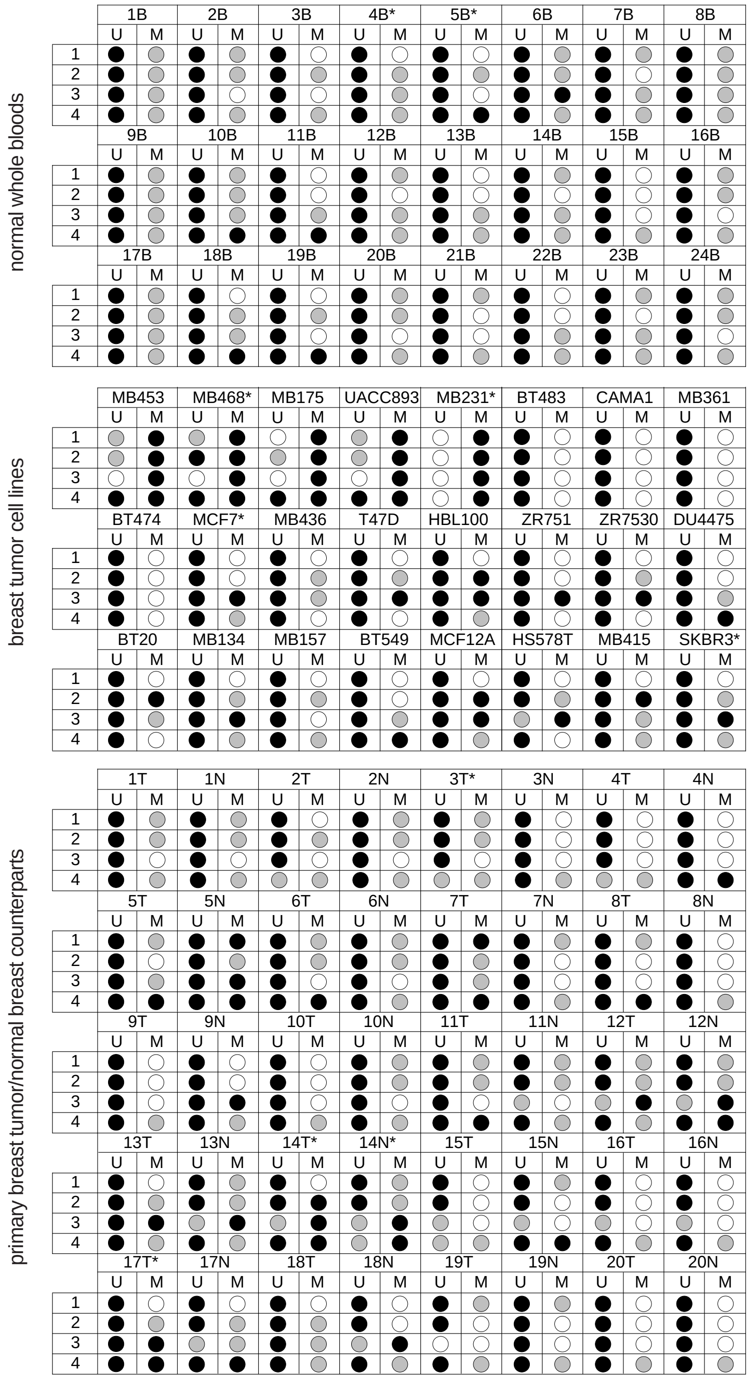
normal whole bloods
1B 2B 3B 4B* 5B* 6B 7B 8B
9B 10B 11B 12B 13B 14B 15B 16B
17B 18B 19B 20B 21B 22B 23B 24B
breast tumor cell lines
MB453 MB468* MB175 UACC893 MB231* BT483 CAMA1 MB361
BT474 MCF7* MB436 T47D HBL100 ZR751 ZR7530 DU4475
BT20 MB134 MB157 BT549 MCF12A HS578T MB415 SKBR3*
primary breast tumor/normal breast counterparts
1T 1N 2T 2N 3T* 3N 4T 4N
5T 5N 6T 6N 7T 7N 8T 8N
9T 9N 10T 10N 11T 11N 12T 12N
13T 13N 14T* 14N* 15T 15N 16T 16N
17T* 17N 18T 18N 19T 19N 20T 20N
U M
1 2 3 4
