# Supplementary material for: Aberrant CBFA2T3B gene promoter methylation in breast tumors
Source: Mol Cancer. 2004 Aug 10;3:22. doi: 10.1186/1476-4598-3-22 (PMC516017; doi:10.1186/1476-4598-3-22)

Additional file 3. Real-time MSP demonstrating aberrant CBFA2T3B promoter CpG methylation in breast tumor cell lines

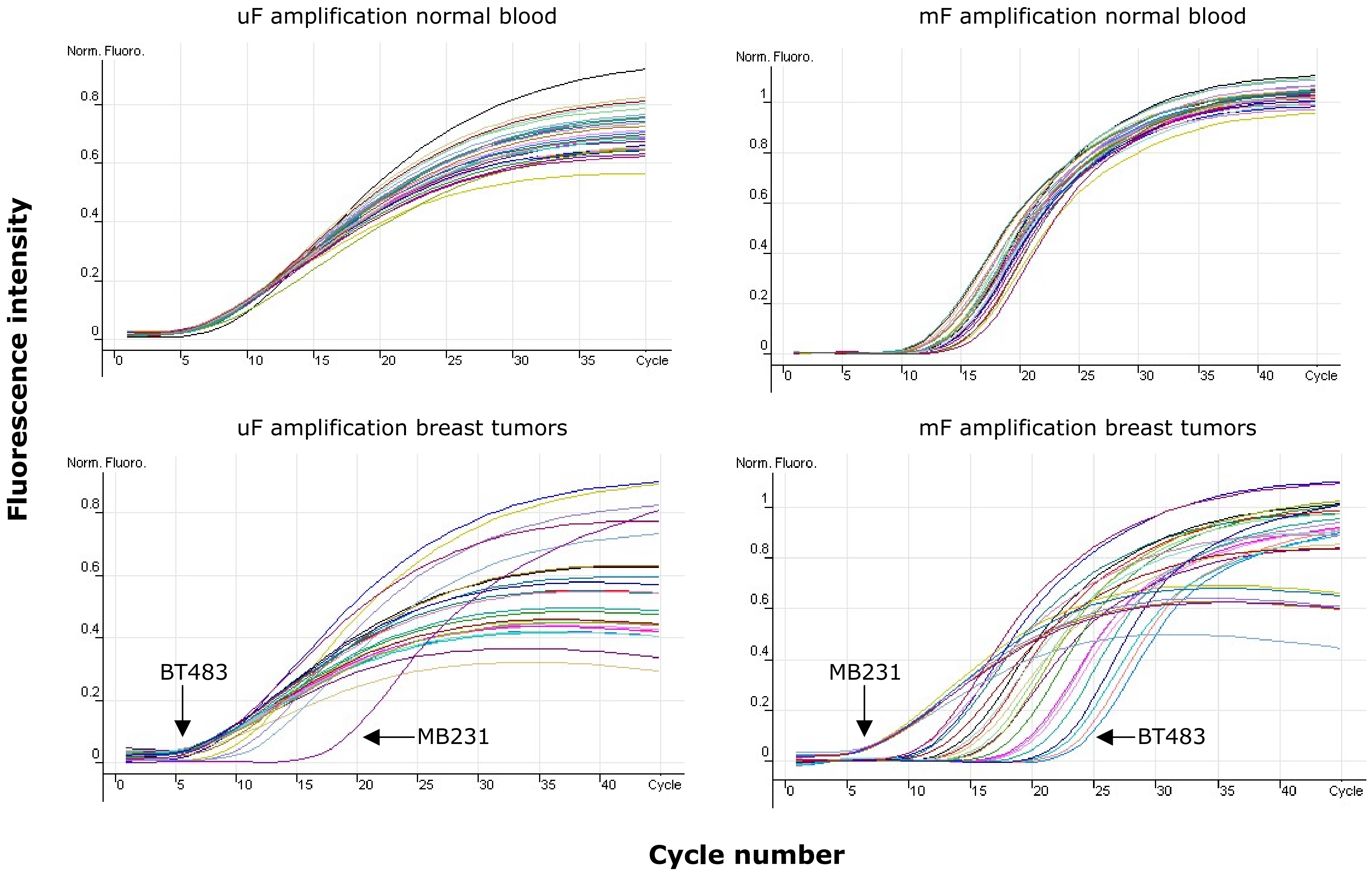

Supplement: Additional File 3 — CBFA2T3B promoter methylation levels assayed using second-round real-time MSP CBFA2T3B promoter methylation levels were assayed using second-round real-time MSP. The raw data methylation levels in normal whole blood samples and breast tumor cell lines are shown. The y-axis represents the fluorescence detection scale and the x-axis represents the CT of amplification. Second round real-time MSP was performed on a bisulfite sequencing amplicon using internal forward primers to detect for either unmethylated (uF) or methylated (mF) cytosines at the Sp1 CpG sites shown in Figure 1C. The CT for methylated amplification in breast tumor cell lines was highly aberrant compared to normal blood samples. Note the late unmethylated CT obtained in MDA-MB-231 compared the early hypermethylated CT. In contrast, BT-483 shows an early unmethylated and late hypomethylated CT (pdf file). [file 1476-4598-3-22-S3.pdf]
