# Supplementary material for: Aberrant CBFA2T3B gene promoter methylation in breast tumors
Source: Mol Cancer. 2004 Aug 10;3:22. doi: 10.1186/1476-4598-3-22 (PMC516017; doi:10.1186/1476-4598-3-22)

Additional file 4. Real-time MSP melt curve analysis demonstrating aberrant CBFA2T3B promoter CpG methylation profiles in breast tumor cell lines

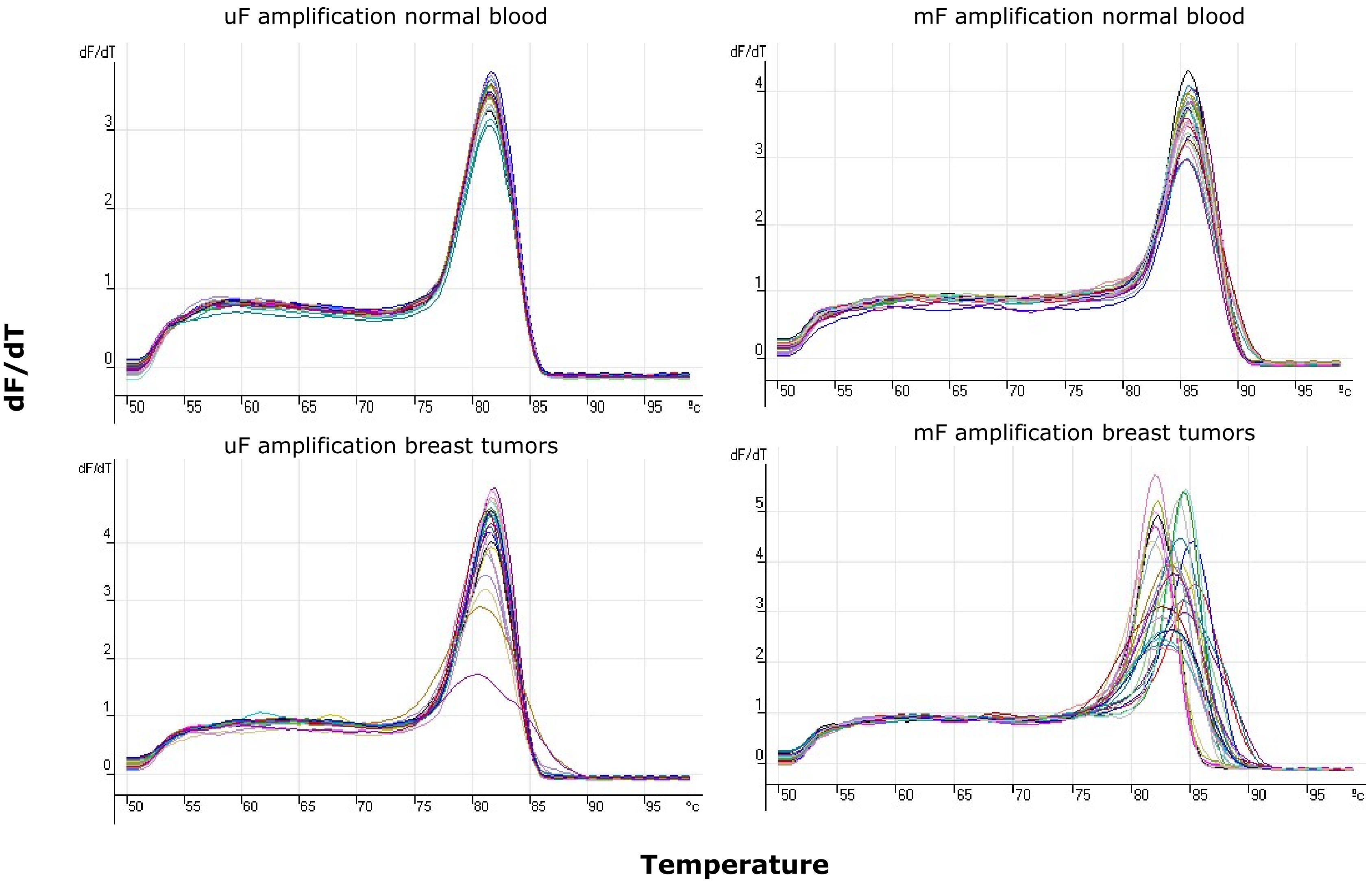

Supplement: Additional File 4 — CBFA2T3B promoter methylation melt curve analysis CBFA2T3B promoter methylation melt curves were examined following second-round real-time MSP. Raw data melt curves of second round amplicons in normal blood samples and breast tumor cell lines are shown. Curves were calculated from the negative derivative in fluorescence over temperature versus temperature (-dF/dTm versus Tm). Normal blood samples displayed consistent peak levels for both the unmethylated and methylated mlcls. In contrast, breast tumor cell lines displayed highly aberrant peak levels depicted by broad melt transitions and heterogeneous melt curves reflective of the aberrant concentration and composition of 5-methylcytosines (pdf file). [file 1476-4598-3-22-S4.pdf]
