# Supplementary figures and images for: Aberrant CBFA2T3B gene promoter methylation in breast tumors
Source: Mol Cancer. 2004 Aug 10;3:22. doi: 10.1186/1476-4598-3-22 (PMC516017; doi:10.1186/1476-4598-3-22)

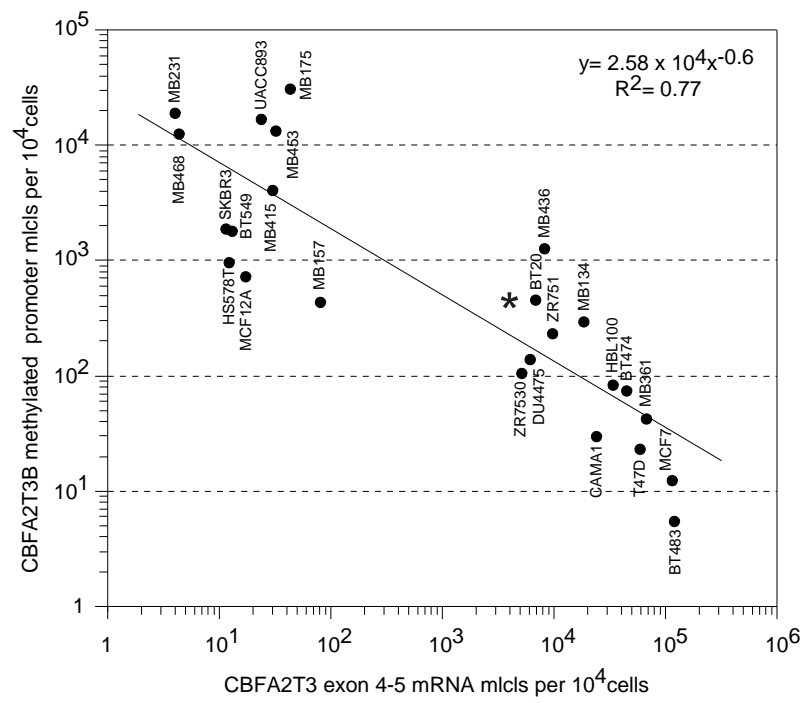

Supplement: Additional File 5 — CBFA2T3B promoter methylation levels versus gene expression Methylation indices were calculated as methylated promoter mlcls per 104 cells for breast tumor cell lines with pre-determined 16q24.3 DNA mlcls per cell and plotted against CBFA2T3 mRNA mlcls per 104 cells. The y-axis represents methylation levels assayed using real-time MSP and the x-axis represents expression levels assayed using real-time RT-PCR. Both data sets are shown on a log scale. Each black circle represents a different breast tumor cell line. The asterisk marks the median methylation and median gene expression levels. The median methylation index of 0.02 (i.e. 2 mlcls or alleles methylated in 100) is calculated from the median methylation level of 450 methylated alleles per 104 cells divided by the number of unmethylated 'active' alleles in the 104 cells or 20,000 alleles, i.e. [450 ÷ (20,000 - 450) = 0.02]. The median gene expression index of 0.2 (i.e. 20 mRNA mlcls expressed in 98 'active' alleles) is calculated from the median expression level of 4,500 mRNA mlcls per 104 cells divided by the number of unmethylated 'active' mlcls in the 104 cells, i.e. (4,500 ÷ 19,550 = 0.2). This calculation equates to approximately 4–5 mRNA mlcls expressed per 10 cells and suggests that the CBFA2T3B gene is largely transcriptionally inert. The remaining active alleles may be trans-factor dependent for expression. An inverse correlation between promoter methylation and expression levels per population of cells was established (r2 = 0.77; r = -0.9, P < .0001). In hypermethylated MDA-MB-231, approximately 17,000 promoter mlcls were methylated (i.e. mi = 0.85 as 17,000 in 20,000 are methylated) and 4 mRNA mlcls expressed per 104 cells. In hypomethylated BT-483, approximately 5 promoter mlcls were methylated (mi = 0.0002) and 120,000 (± 40,000) mRNA mlcls expressed per 104 cells. This elevated expression equates that 12 (± 4) mRNA mlcls per cell are expressed from an estimated four-promoter mlcls per cell (i.e. f [file 1476-4598-3-22-S5.pdf]

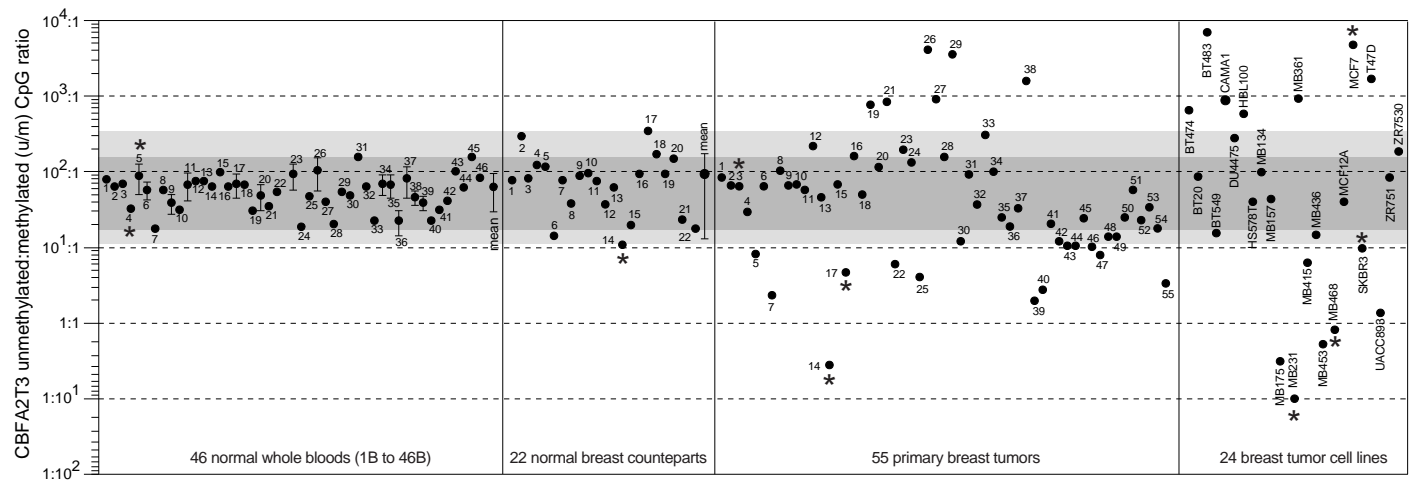

Supplement: Additional File 6 — CBFA2T3B promoter methylation levels assayed using second-round real-time MSP The methylation levels in normal whole blood samples, normal breast counterparts, primary breast tumors and breast tumor cell lines were assayed at the Sp1 site shown in Figure 1C and plotted as absolute methylation ratios (u/m). All samples are labeled corresponding in part to Additional file 2. The asterisks indicate the samples examined by bisulfite sequencing. The gray highlights indicate the normal blood and normal breast counterpart 'full' methylation ranges. Basal methylation ratios in normal bloods averaged 60:1 (cumulative mean) unmethylated to methylated CBFA2T3B promoter mlcls. This average coincided with conventional MSP band intensities of 100:1 based on pUC19 DNA/MspI marker concentrations (see Figure 3) and the median methylation index of 0.02 (i.e. 2 mlcls in 100 are methylated). Normal blood ratios ranged 20:1 to 160:1 unmethylated to methylated mlcls. Normal breast counterparts were similar to normal bloods averaging 100:1 but with a larger range of 10:1 to 350:1. Relative to the normal samples, breast tumors displayed highly aberrant methylation ratios clearly resolved by second-round real-time MSP. 75% of breast tumor cell lines were aberrantly methylated outside the full range of normal blood basal methylation. 58% were outside the range of normal breast. Half of the aberrations were either hypo or hypermethylated relative to both normal blood and normal breast. Similar to cell lines, 51% of primary breast tumors were aberrantly methylated relative to normal blood. 35% were aberrant relative to normal breast (i.e. 24% were hypermethylated and 11% hypomethylated). Aberrant methylation ratios ranged from 1:10 in hypermethylated MDA-MB-231 to 7,000:1 in hypomethylated BT-483 (pdf file). [file 1476-4598-3-22-S6.pdf]
