# Supplementary material for: Aberrant CBFA2T3B gene promoter methylation in breast tumors
Source: Mol Cancer. 2004 Aug 10;3:22. doi: 10.1186/1476-4598-3-22 (PMC516017; doi:10.1186/1476-4598-3-22)
Supplement: Additional File 7 — Primers, probes and annealing temperatures used (pdf file). [file 1476-4598-3-22-S7.pdf]

Amplicon (bp, ^{o}C)	Forward primer (5'-3')	Probe (5'-3')	Reverse primer (5'-3')
CYPA-1 (114, 60)		FAM-acaccacatgcttgccatccaaccact-BHQ1	
	aatgctggacccaacacaaatg		cacaatattcatgccttctttcacttt
CBFA2T3B (75, 60)		FAM-cgtcccacccaccctgagccctac-TAMRA	
	cagccgcccctgagtcgt		gcacactcgcgactcggt
CBFA2T3A (77, 60)		FAM-ctgctccttccagcttggtccccgc-TAMRA	
	cgaggaaacacatgcccagt		cctcctcccaggcagctc
CYPA-2 (355, 60)	ggcaaatgctggacccaacacaaa		ctaggcatgggagggaacaaggaa
ATP5A (270, 60)	ttgcggaggaacattggtg		tcttcaggcctggggttttt
CBFA2T34/5 (80, 60)	gggcctggtgaactcgacattgac		acggccgcagagggaagttggt
SYK (373, 60)	aggccaatgaccccgctctta		tgggtctgcgccttgtagtagttt
1F/1Rm (116, 60)	cggcgttagaagatagcgcgtagtcg		atccgacgaacgacgaacgacg
1F/1Ru (133, 52)	tttgagtttggtgtggtgttagaagatagtgtg		aaaaatccaacaaacaacaaacaaca
2F/2Rm (123, 60)	ttataggcgcggcggttggattcg		gaccgcctaaaccatacgaaaactcg
2F/2Ru (135, 52)	tataggtgtggtggttggatttg		aaacacacaaaacaaccacctaaaccatacca
3F/3Rm (198, 60)	gcggttttaaagttgcgagttcg		aacgcgccgcctcctacgaaaaacg
3F/3Ru (188, 52)	agtttgtgggtgttgattgttgtg		ccacctcctacaaaaaacaacaca
4F/4Rm (112, 60)	gcgcgattttgtcggttacgtacg		aaaaaaatcgctccttcgaccgccg
4F/4Ru (115, 52)	ggggagtgtgatttttgttggttatgtatg		aaaatcactccttcaaccaccacccccca
1BF/1BR (365, 52)	tttttgtaggtggatatgaaa		acacccacctcrcaacaa
2BF/2BR (310, 52)	ttgttgygaggtgggtgt		acctaaaaaaaaaactcccc
3BF/3BR (346, 52)	ggttttaaagttgygagtt		ctaaaccataccraaaact
4BF/4BR (477, 52)	ggggagtttttttttaggt		acacttacaatcraaaaatcc
mF/2BR (187, 56)	tttgcgttttttcgcgtcg		acctaaaaaaaaaactcccc
uF/2BR (187, 52)	tttgtgtttttttgtgttg		acctaaaaaaaaaactcccc
